# Role of hydrogen sulfide donors in cancer development and progression

**DOI:** 10.7150/ijbs.47850

**Published:** 2021-01-01

**Authors:** Ebenezeri Erasto Ngowi, Attia Afzal, Muhammad Sarfraz, Saadullah Khattak, Shams Uz Zaman, Nazeer Hussain Khan, Tao Li, Qi-Ying Jiang, Xin Zhang, Shao-Feng Duan, Xin-Ying Ji, Dong-Dong Wu

**Affiliations:** 1 Henan International Joint Laboratory for Nuclear Protein Regulation, School of Basic Medical Sciences, Henan University, Kaifeng, Henan 475004, China; 2 School of Stomatology, Henan University, Kaifeng, Henan 475004, China; 3 Department of Biological Sciences, Faculty of Science, Dar es Salaam University College of Education, Dar es Salaam 2329, Tanzania; 4 Kaifeng Municipal Key Laboratory of Cell Signal Transduction, Henan Provincial Engineering Centre for Tumor Molecular Medicine, Henan University, Kaifeng, Henan 475004, China; 5 Faculty of Pharmacy, The University of Lahore, Lahore, Punjab 56400, Pakistan; 6 School of Life Sciences, Henan University, Kaifeng, Henan 475004, China; 7 Institute for Innovative Drug Design and Evaluation, School of Pharmacy, Henan University, Kaifeng, Henan 475004, China; 8 Kaifeng Key Laboratory of Infection and Biological Safety, School of Basic Medical Sciences, Henan University, Kaifeng, Henan 475004, China

**Keywords:** Hydrogen sulfide, H_2_S donors, Cancer, Signaling pathways, Cellular processes

## Abstract

In recent years, a vast number of potential cancer therapeutic targets have emerged. However, developing efficient and effective drugs for the targets is of major concern. Hydrogen sulfide (H_2_S), one of the three known gasotransmitters, is involved in the regulation of various cellular activities such as autophagy, apoptosis, migration, and proliferation. Low production of H_2_S has been identified in numerous cancer types. Treating cancer cells with H_2_S donors is the common experimental technique used to improve H_2_S levels; however, the outcome depends on the concentration/dose, time, cell type, and sometimes the drug used. Both natural and synthesized donors are available for this purpose, although their effects vary independently ranging from strong cancer suppressors to promoters. Nonetheless, numerous signaling pathways have been reported to be altered following the treatments with H_2_S donors which suggest their potential in cancer treatment. This review will analyze the potential of H_2_S donors in cancer therapy by summarizing key cellular processes and mechanisms involved.

## Introduction

Cancer has remained as one of the most investigated diseases in scientific research, and many potential therapeutic targets have been discovered. However, developing the most effective and highly efficient combination therapy against the extremely resistant metastatic cancer cells is still a challenge. In 2018, the worldwide estimation of cancer new cases was 18.1 and the mortality numbers were 9.6 million 1, which is an increase of more than 28% and 17% respectively from the 2012 report 2. This enormous increase in new cases demands for the new and effective therapeutic option for the disease. Cellular activities such as autophagy, apoptosis, migration and proliferation that are known to be altered in cancer cells serve as the main therapeutic targets 3-5. Therefore, the identification of potential compounds that can effectively regulate these activities to bring about anti-cancer properties is essential in determining potential treatments.

Hydrogen sulfide (H_2_S) is the third gaseous neurotransmitter to be associated with the regulation of pathophysiological processes, next to carbon monoxide (CO) and nitric oxide (NO) 6, 7. H_2_S is initially synthesized in the cells by the enzymatic reactions involving cystathionine beta-synthase (CBS), 3-mercaptopyruvate sulfurtransferase (3-MPST) and cystathionine gamma-lyase (CTH/CSE) enzymes 6, 8, 9. H_2_S plays a vital role in the establishment and progression of multiple health disorders ranging from non-cancerous 10, 11 to cancerous 12, 13. In cancer, H_2_S participates in both the suppression 12, 14-17 and progression of the disease 13, 18.

H_2_S donors are naturally or synthetically derived H_2_S-releasing compounds. Some of the commonly used H_2_S donors include sodium polysulthionate, 1,2-dithiole-3-thiones, modified non-steroidal anti-inflammatory drugs, sulfide-containing salts, sodium hydrosulfide (NaHS), sodium sulfide, garlic-derived natural sulfides, allyl methyl sulfide, dipropyl disulfide, diallyl sulfide (DAS), diallyl disulfide (DADS), diallyl trisulfide (DATS), Lawesson's reagents, morpholin-4-ium-4-methoxyphenyl(morpholino) phosphinodithioate (GYY4137) and its derivatives, diclofenac derivative, 4-(3-thioxo-3H-1,2-dithiol-5-yl)phenyl ester-2-[(2,6-dichlorophenyl) amino]-benzeneacetic acid, phenol derivative, 5-(4-hydroxyphenyl)-3H-1,2-dithiole-3-thione (ADT-OH), mesalamine derivative, 5-amino-2-hydroxy-benzoic acid 4-(5-thioxo-5H-[1,2]dithiol-3-yl)-phenyl ester hydrochloride, naproxen derivative, 2-(6-methoxynapthalen-2-yl)-propionic acid 4-thiocarbamoyl phenyl ester (ATB-346), and the mitochondria-specific H_2_S donor, 10-oxo-10-(4-(3-thioxo-3H-1,2-dithiol-5-yl)phenoxy)decyl) triphenylphosphonium bromide 19-21. In a dose and time-dependent manner, these compounds can further be categorized into fast-releasing donors 22, 23 and slow-releasing donors 14, 24, 25. Generally, the efficiency of these drugs is determined by parameters such as solubility, toxicity, and formation of byproducts. The donation of H_2_S occurs through numerous chemical reactions such as hydrolysis, thiol activation, bicarbonate activation, light activation, carboxylesterases metabolism 20.

Despite the recent developments in the field, the role of H_2_S donors in cancer development and progression is still disputable. To date, numerous H_2_S releasing drugs with different properties, donating mechanisms, and capabilities have been synthesized for experimental use. With the exception of their molecular targets, the anti-cancer effects of H_2_S donors have been extensively reviewed 26-29. Herein, we will summarize recent findings on the roles of H_2_S donors in cancer management by illuminating their molecular targets, mechanisms involved and their downstream effects in cellular activities. We further highlight the therapeutic potential of these donors individually or in combination with other drugs.

### Methodology for literature selection

Relevant articles were searched using PubMed, Google, Google Scholar, and Research Gate with the keywords: 'Hydrogen sulfide and cancer', 'Hydrogen sulfide donors and cancer', and by using specific H_2_S drug names (e.g. 'Diallyl trisulfide and cancer'). In addition to the relevancy of the title and abstracts, articles were selected for inclusion based on the year of publication which was mainly between 2015 and 2020. Though, few older articles were also included to strengthen the knowledge. The articles selected have been cited accordingly.

## Physiological and pathological effects of H_2_S

H_2_S regulates numerous physiological processes in the body. Both endogenous and exogenous-derived H_2_S have been shown to participate several physiological activities such as muscle relaxation, water regulation, cytoprotection, and inflammation 30-33. Low levels of H_2_S and the downregulation of its producing enzymes are familiar events in cancer cells. The analysis of tissue samples from hepatocellular carcinoma (HCC) patients reveals a significant reduction in CBS levels compared to their surrounding non-cancerous tissues and the decrease could be correlated with poor prognosis 34. Further downregulation of the CBS enzyme using an inhibitor CH004 induces anti-cancer effects via ferroptosis, an iron-dependent programmed cell death in HepG2 cells 35. Unlike CBS, CSE is upregulated in many cancer types including HCC, gastric and breast cancer, where its endogenous inhibition also has anti-carcinogenic effects 17, 36, 37. Alternatively, studies have shown that the supplementation of H_2_S using donors can also induce strong cytoprotective effects in many cancer types 14, 37-39. Despite the lack of evidence on the normal range of H_2_S in different cells and the activities leading to its alteration in cancer cells, the available data suggest that only a certain amount of H_2_S is required for maintaining cellular activities and any adjustment resulting from either increasing or decreasing the level has an enormous impact on cellular activities involved in cancer modulation.

## Mechanisms involved in H_2_S-mediated cancer modulation

One of the primary mechanisms involved in the H_2_S-induced cancer regulation is through its interaction with cellular transporter/channels. As a neurotransmitter, H_2_S has been shown to interact with cell transporters 40 and ion channels 41-43, resulting in the downstream regulation of cellular activities 43-45. It has been revealed that the treatment of HEK293 cells with 1 µM-1 mM NaHS inhibits voltage-gated T-type specifically Cav3.2 channels by promoting extracellular binding of zinc (Zn^2+^) 46. At the downstream, the suppression of T-type channels can result in enhanced antitumor activities and improved sensitivity of cancer cells to drugs 47-49. Studies also show that H_2_S donors can trigger the activation of cellular transporters including ATP-binding cassette transporter A1 (ABCA1) 40 and glutamine transporter-1 (GLT-1) 50 by promoting nuclear translocation of peroxisome-proliferator-activated receptor alpha and inhibiting the extracellular signal-regulated kinase 1/2 (ERK 1/2) respectively. However, depending on the cancer type, the activation of ABCA1 and GLT-1 can either have inhibitory or promoting effects 45, 51-53. Moreover, by interacting with GLT-1, H_2_S donors can directly participate in modulating aerobic glycolysis (Warburg effect) which is a metabolic-hallmark of most cancer cells. Therefore, the impact of the activation of the above transporters by H_2_S donors in cancer should be further examined to clarify the mechanism and the subsequent responses. Besides, H_2_S can also interact with insulin receptors and toll-like receptors 4 (TLR4), resulting in the inhibition of phosphoinositide 3-kinase (PI3K)/AKT/mTOR 12, nuclear factor-kappa B (NF-кB) 54 and signal transducer and activator of transcription-3 (STAT-3) 55 pathways together with their corresponding cellular responses.

Another key mechanism involved in the regulation of cellular activities is the functional interconnection between H_2_S and other gasotransmitters. This potential link between H_2_S, CO, and NO serves as a crucial mechanism exploited by H_2_S donors in regulating cancer activities. Exclusively, exogenous treatment with donors of either of the three gasotransmitters can reduce cancer progression by facilitating reactive oxygen species (ROS) tolerance, anti-proliferative and pro-apoptotic responses in human breast cancer 56-58. Moreover, a recent study indicates that treatment with either NO, CO, or H_2_S donors (namely S-nitroso-N-acetyl-D,L-penicillamine, carbon monoxide releasing molecule-A1, and GYY4137, respectively) promotes anti-carcinogenic activities in colon cancer cells HCT116 in a dose-dependent manner by regulating AKT, cyclic guanosine monophosphate (cGMP)/VASP, and P44/42 mitogen activated protein kinase (MAPK) pathways 16. With respect to the link, study shows that the treatment of cancer cells with DAS, DATS, and diallyl tetrasulfide can significantly reduce tumor growth mechanistically by upregulating heme oxygenase-1 (HO-1) expressions through MAPK and PI3K-mediated activation of nuclear factor erythroid-2 related factor-2 (Nrf-2) pathway 59-61. The downstream effect of HO-1 is the catalysis of heme to biliverdin, a reaction that releases CO as a byproduct 62. It is also worth noting that treatment with CO donor photo-CORM [Mn (CO)_3_] reduces the antioxidant status by inhibiting the bioactivity of H_2_S producing enzyme CBS in breast cancer cells 63. Moreover, it has been reported that both the treatment with 20-100 µM NaHS following the inhibition of long non-coding RNA sONE (an endothelial NO regulator) on breast cancer cells MDA-MB-231 for 72 hours, or the co-treatment of NaHS with 40-100 µM doses of NO donor diethylenetriamine NO adduct (DETA/NO) can reverse the antitumor effects of NaHS by regulating cGMP pathways 64. Similarly, NaHS treatment also increases the production of NO and its associated cytoprotective effects in a pH-dependent manner in murine brain homogenate and L1210 leukemia cells conceivably via S-nitrosothiol-signaling pathway 65. In summary, the above data indicate the interaction between H_2_S, CO, and NO to play a significant part in regulating cancer activities, which is in addition to its interaction with cell receptors and transporters.

## Role of H_2_S donors in cancer

The response of cancer cells to H_2_S donors varies substantially depending on the donor type, concentration and cancer types. Here, we will discuss the effects of these donors in cancer by illuminating cancer-promoting activities (including immune response, gene transcription and translation, and cell growth), cancer inhibitory events (such as cell cycle arrest, autophagy, and apoptosis), anti-cancer drug sensitivity and *in vivo* tumor growth.

### Cancer suppressing activities

#### H_2_S donor regulates immune responses

The immune system plays an important role in maintaining normal cellular activities. In the initial stages of cancer, it helps in tumor inhibition by removing immunogenic cancer cells, however, in later stages it is manipulated to facilitate cancer growth and progression 66. One of the ways in which cancer cells escape from being destroyed by the immune system is through regulatory T cells (Tregs) 67. By regulating the transforming growth factor-beta (TGF-ꞵ), Tregs can markedly promote epithelial-mesenchymal transition (EMT) of HCC cells 68 and suppress the cytotoxicity of the expanded tumor-specific CD8 T cells 69. High frequency of FOXP3^+^ CD4^+^ CD25^+^ regulatory T cells has been shown to facilitate cancer progression in pancreatic ductal adenocarcinoma 70 and HCC 71. Though, the binding of ten-eleven translocation methylcytosine dioxygenases 1 and 2 (Tet 1 and 2) to FOXP3^+^ can help to maintain the stability and functions of Tregs 72, 73. A substantial reduction in the expressions of Tet genes has been reported in several cancer types including colon and breast cancer and their upregulation promotes tumor-suppressing effects 74-77. It has been revealed that treatment with H_2_S donors can stimulate DNA demethylation and Tregs-associated immune responses in CD4^+^ T cells by increasing the expressions of Tet 1 and Tet 2 genes via the sulfuration of nuclear transcription factor Y subunit beta in H_2_S-deficient mice model 78. However, whether H_2_S donors can deregulate Tet 1 and 2 in cancer types including ovarian cancer 79 and where the Tet 1 and 2 genes are overexpressed need to be further investigated.

Peroxynitrite, an extremely reactive compound formed from the reaction between NO and superoxide radicals, is known to promote DNA damage and trigger autoantibody in cancer patients 80. Filipovic et al. reports that H_2_S treatment can effectively prevent the effects of peroxynitrite by reacting with the compound under inflammatory conditions to form sulfinyl nitrite, an NO-releasing compound 81. Furthermore, treatment with NaHS protects glomerulus mesangial and Jurkat cells against antibody-induced cell lysis and apoptosis by sulfurating the humoral effector molecules thereby reducing antibody binding ability 82. NaHS also suppresses the activation of the complement alternative pathway (AP), an important effector molecule of innate immunity. Regardless, the effect of the inhibition is still uncertain since both overactivation and underactivation of the pathway have been observed in cancer 83, 84. In addition, whether the event could impair immune response and expose patients to other infections needs further exploration. Collectively, these data imply an essential role of H_2_S in regulating immune activities and associated diseases including cancer through the regulation of key immune regulators.

#### H_2_S donor mediates gene transcription and translation

The transcription and translation factors are vital entities that regulate the generation of RNA molecules and amino acid sequences respectively 85, 86. The dysregulation of transcription and translation factors are common events in cancer cells 87, 88. It has been shown that treatment with H_2_S donors can effectively regulate numerous transcription factors including NF-кB 32, STAT-3 55, and Nrf-2 89-91 that are involved in inflammation, apoptosis, and oxidative stress events. Moreover, GYY4137 treatment can significantly downregulate the expression of transcription factor Krüppel-like factor 5 (KLF-5) 92, which in turn regulates the SRY-box transcription factor-4 93 and NF-кB 94.

Besides, H_2_S plays a key role in regulating the post-translation modification of protein via sulfuration 95. In mouse embryonic fibroblast cells (MEF) and HeLa cells, treatment with 100 µM NaHS significantly increases the phosphorylation of eukaryotic translation initiation factor 2 subunit alpha (eIF2α) partially through the suppression of protein phosphatase 1c as a result of its persulfidation at cysteine (Cys)-127 96. Even though the phosphorylation of eIF2α decreases protein synthesis, the incident can result in the promotion of cell migration and ultimately cancer metastasis 97. Hence, the interaction between H_2_S and eIF2-α, and its implication in carcinogenic activities needs to be further investigated. Alternatively, treatment with NaHS or GYY4137 could protect against apoptotic and inflammatory responses by sulfurating the p65 subunit of NF-кB at Cys-38 in monocyte/macrophage THP-1 cells, RAW macrophages and human embryonic kidney (HEK-293) cells 98, 99. In addition, NaHS, GYY4137 or DATS treatments improves antioxidant status through post-translation modification of Kelch-like ECH associated protein 1 (Keap 1), a redox-sensitive protein with the first two donors mediating the sulfuration at Cys-151 100, 101 and the later at Cys-288 residues 100, 102. Also, a report by Hourihan et al. reveals the formation of a disulfide bond and sulfuration of Keap1 at Cys-226 and Cys-613 following NaHS treatment in COS 1 cells 103. The resulting sulfuration-induced inhibition of Keap 1 exerts anti-proliferation responses through the activation of the Nrf-2 antioxidant pathway. Meanwhile, in hypertensive rats, GYY4137 treatment attenuates the transcription activities of KLF-5 via S-sulfuration of specificity protein-1 (SP-1) at Cys-664 92, thus stimulating anti-cancer responses 104. Generally, H_2_S donors interact with key transcription and translation factors involved in cellular activities, most of which are considerably dysregulated in cancer cells which confirm their potential in cancer treatment.

#### H_2_S donor blocks cell cycle

Targeting cell cycle-mediating processes have been identified as a viable therapeutic option for treating numerous disorders including neurodegenerative disorders 105 and cancer 106. Cell cycle arrest is an important event responsible for evaluating cellular damages and initiating apoptosis 107. Cell cycle contains 4 main phases, namely Gap 1 (G1), Synthesis (S), Gap 2 (G2), and Mitosis (M) phase, that are closely monitored by the G1/S and G2/M checkpoints 108. Besides, cells exit the cycle temporarily or permanently by entering the quiescent/resting phase (G0). The activation of cell cycle regulators, cyclin-dependent kinases (CDK) helps to switch the cycle on and off 109. Both cell cycle checkpoints 110 and CDKs 111 have been established as the vital targets in cancer treatment. The administration of GYY4137 induces cell cycle arrest at G1/S checkpoint in HCC cells 55, and S-G2/M phases in colorectal cancer 15 and breast cancer cells 14. Moreover, NaHS treatment prevents cell cycle progression by triggering G0/G1 arrest in breast cancer 112 and non-small cell lung cancer (NSCLC) 113.

It has also been revealed that treatment with DATS can promote DNA damage and G2/M arrest through the activation of ataxia telangiectasia mutated kinase and upregulation of nuclear exportation of cell division cycle 25C protein in thyroid and bladder cancer 114, 115, and by delaying nuclear translocalization of CDK1 in prostate cancer 116. Moreover, DATS increases the expressions of intercellular cyclins (A2 and B1), apoptotic markers (Bcl-2-associated X protein (Bax), p53, cleaved caspase 8, 9, and cytochrome c) and histone 3 phosphorylation in gastric cancer 117, 118. In osteosarcoma cells, DATS treatment mediates ROS-dependent G0/G1 arrest by suppressing the protein expressions of cyclin D1 and increasing those of p21 and p27 119. The protective effects of DATS is induced via the activation of MAPK and AMP-activated protein kinase (AMPK) as well as the inhibition of PI3K/Nrf-2/AKT pathways 117-119.

Besides, treatment with DADS also induces G2/M arrests in pancreatic 120, ovarian 121, and colon cancer cells 122 by promoting DNA-damage and activating mitochondria apoptotic pathways. In summary, H_2_S donors regulate cell cycle progression by interacting with cell cycle regulators and checkpoints, suggesting the potential of these compounds in cancer treatment.

#### H_2_S donor attenuates cell proliferation and viability

**Figure 1:**
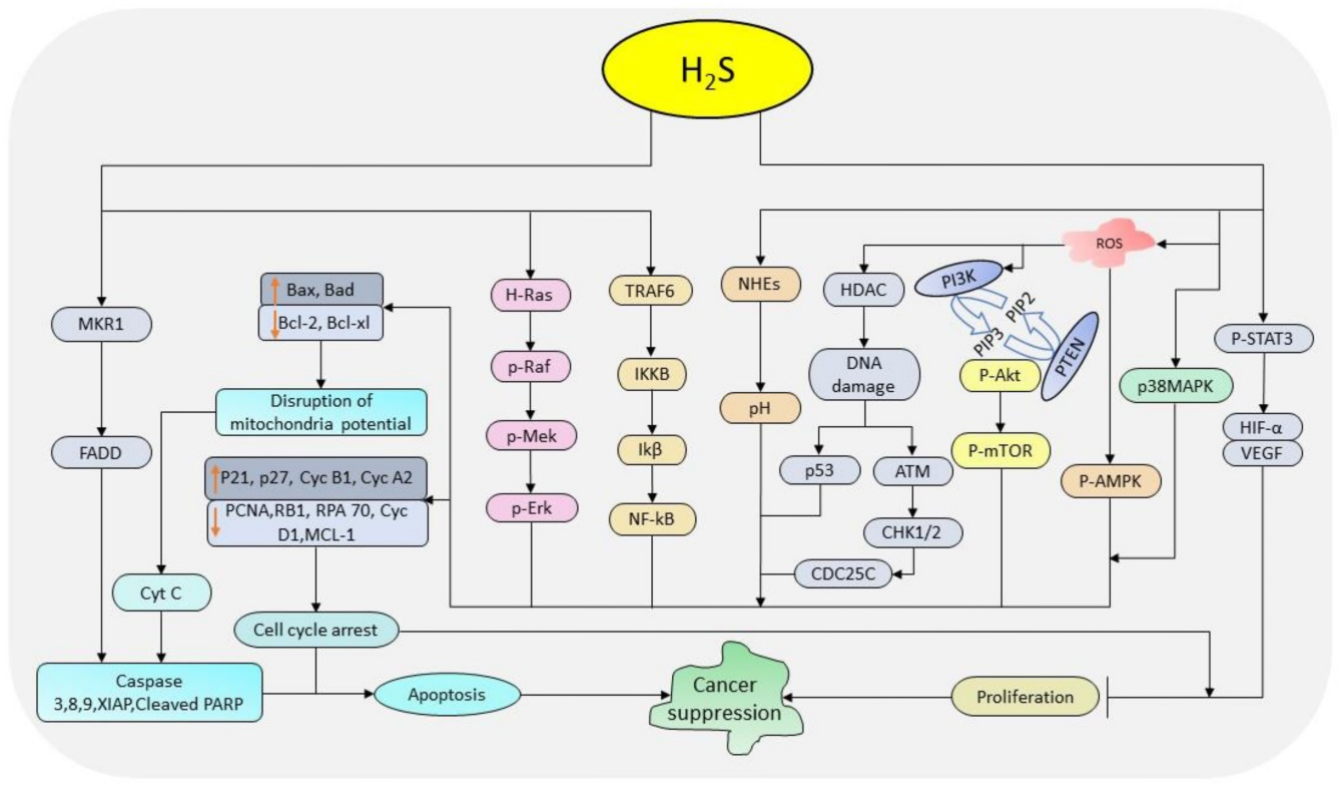
The diagrammatic illustration of the pathways regulated by H_2_S donors in cancer suppression. H_2_S donors regulate STAT-3, MAPK, AMPK, PI3K-mTOR, p53, ATM-CHK1/2-CDC25C, NF-кB, and Ras-Raf-Mek-Erk pathways together with the intercellular pH and cell cycle regulators resulting into the inhibition of cell cycle progression and promotion apoptosis. (STAT-3: signal transducer and activator of transcription-3; MAPK: mitogen-activated protein kinase; AMPK: AMP-activated protein kinase; PI3K: phosphoinositide 3-kinase; mTOR: mammalian target of rapamycin; p53: tumor protein 53; ATM: ataxia telangiectasia mutated kinase; CHK1/2: checkpoint kinases 1/2; CDC25C: cell division cycle 25 C protein; NF-кB: nuclear factor-kappa B; Raf: rapidly accelerated fibrosarcoma; ERK: extracellular signal-regulated kinase)

Cancer cells are characterized by abnormal/uncontrolled proliferation resulting into their accumulation. The link between cell cycle regulators and signaling pathways plays a crucial role in regulating cell proliferation and viability. H_2_S donors modulate cell proliferation and viability by interacting with the cell cycle regulators and related signaling pathways 118, 119, 123. In colon and breast cancer, GYY4137 treatment attenuates pro-proliferation activities by promoting cell cycle arrest, apoptosis, and necrosis 14, 15. Also, treatment with DATS decreases cell viability and proliferation in gastric cancer 118, osteosarcoma 119, and glioma 123 through the activation of MAPK and suppression of PI3K/AKT cascades, and wingless integrated (Wnt)/beta catenin (ꞵ-catenin) pathways respectively. Concomitantly, treatment with DADS downregulates cyclin D1 and *c-*myc in osteosarcoma 119, and CDK1 and Cyclin B1 in ovarian cancer 121. As a tumor suppressor, NaHS also inhibits the growth of HepG2 cells by regulating PI3K/AKT/mTOR pathway 38 and that of breast cancer MCF-7 cells via p38 MAPK pathway 112. Overall, H_2_S donors regulate cell proliferation and viability in a cell/dose/time-dependent manner by interacting with a wide range of signaling cascades. The above evidences indicate that these donors can serve as a potential candidate in cancer treatment through their ability to regulate key cellular pathways associated with proliferation and viability activities. The summary anti-cancer pathways regulated by H_2_S donors is depicted in Figure 1.

#### H_2_S donor inhibits cell migration and invasion

**Figure 2:**
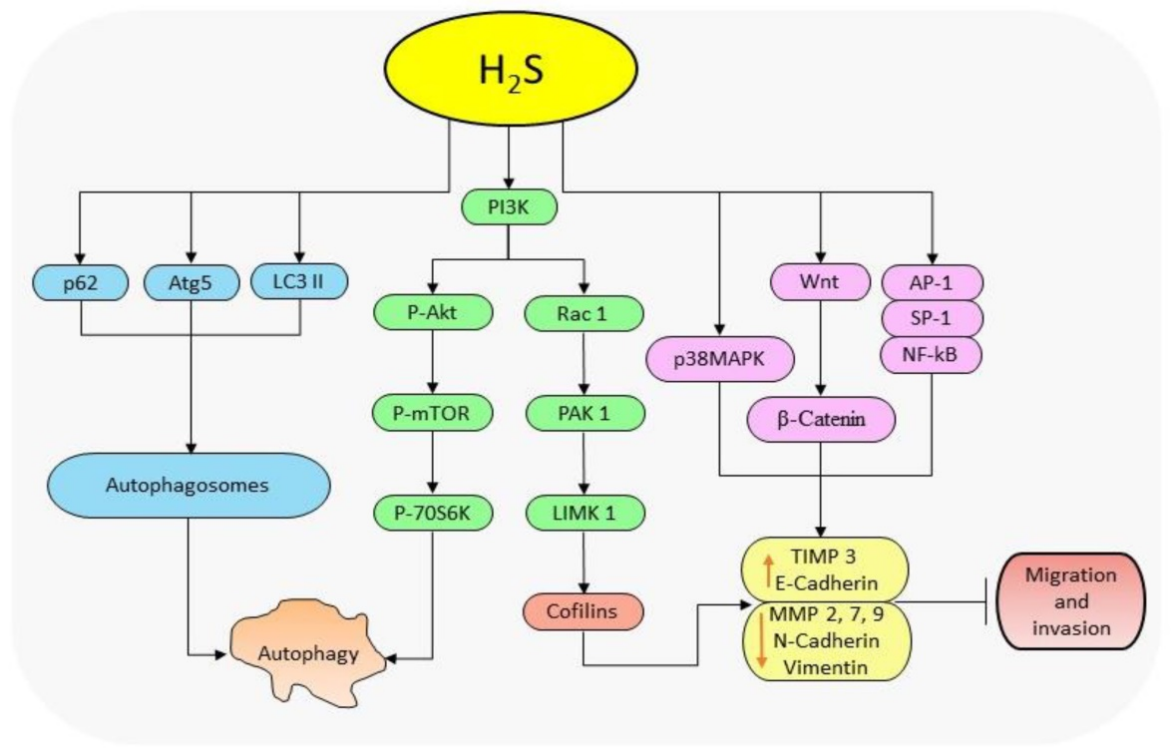
The diagrammatic illustration of the mechanism by H_2_S donors in regulating autophagy and cell migration. H_2_S reduces the levels of p62 and elevates those of Atg5 and LC3-II to facilitate the formation of autophagosomes resulting in autophagy. Also, H_2_S suppresses the protein levels of p-PI3K, p-AKT and mTOR resulting in the downstream regulation of p-70S6K expressions and subsequently autophagy. By regulating PI3K, H_2_S also downregulates the expressions of Rac-1, PAK-1, and cofilin thereby reducing MMP-2, -7, -9, N-cadherin and vimentin levels, and increases TIMP 3 and E-cadherin levels resulting in the inhibition of cell migration. H_2_S further activates Wnt/β-catenin pathway to suppress migration activities, and inhibits NF-қB and MAPK pathways to reduce migration and invasion activities. (p62: nucleoporin 62; Atg5: autophagy-related protein 5; LC3 II: LC3-phosphatidylethanolamine conjugate; PI3K: phosphoinositide 3-kinase; AKT: protein Kinase B; mTOR: mammalian target of rapamycin; p-70S6K: ribosomal protein S6 kinase beta-1; Rac-1: Ras-related C3 botulinum toxin substrate 1; PAK-1: p21-activated kinase 1; MMPs: matrix metalloproteinases; TIMP 3: tissue inhibitor of metalloproteinase-3; MAPK: mitogen-activated protein kinase; NF-кB: nuclear factor-kappa B).

Migration and invasion are key players in cancer metastasis and progression. Under these processes, an individual or a collective cluster/strand/cords of cancer cell(s) detach from the primary tumor, penetrate the stroma of the surrounding tissue, enter the blood vessels, and eventually be transported into other organs 124. The whole process is regulated by a comprehensive group of enzymes including extracellular matrix-degrading enzymes, matrix metalloproteinases (MMPs), adhesive enzymes (E-cadherin), desmosomes, and integrins 125. It has been revealed that treatment of HCC cells with 600-1000 µM NaHS effectively inhibits migration and invasion in a concentration-dependent fashion through the regulation of epidermal growth factor receptor (EGFR)/ERK/MMP-2 and PTEN/AKT pathways 126. Similarly, in thyroid cancer cells, 200 µM NaHS treatment inhibits migration activities by deactivating the PI3K/AKT/mTOR and MAPK pathways 127. In lung cancer A549 cells, treatment with 100 µM NaHS significantly reduces nickel-induced EMT and migration by regulating TGF-ꞵ1 pathway 128. Moreover, NaHS decreases the protein levels of MMP-2 in gastric cancer 39, and EMT-inducing snail (SNAI 1) in breast cancer 112. A previous study conducted using DAS, DADS, and DATS in colon cancer Caco-2 cells indicates a significant reduction in migration-associated proteins MMP-2, -7, and -9 129, with DATS showing the highest inhibition rate followed by DADS and DAS. In colon cancer HT29 cells, DATS prevents the angiogenesis and migration by suppressing vascular endothelial growth factor (VEGF), MMPs and inhibiting p38MAPK, focal adhesion kinase (FAK) and JNK signaling cascades 130. Similarly, DATS reduces the migration and invasion of glioma cells by impeding the activation of Wnt/ꞵ-catenin pathways 123. Besides, treatment with DADS prevents migration and invasion of breast cancer stem cells by suppressing the glycolytic enzyme pyruvate kinase M2-induced activation of the AMPK pathway as well as by increasing and decreasing the levels of E-cadherin and vimentin, respectively 131. The treatment with DADS also inhibits Rac1/PAK1/LIMK1/cofilins signaling cascades in colon and gastric cancer 132, 133. The above data suggest that H_2_S donors regulate migration and invasion activities through their interactions with numerous pathways and enzymes (Figure 2).

#### H_2_S donor promotes autophagy

Autophagy is a key process involved in maintenance of cell homeostasis by degrading damaged organelles and misfolded proteins, thereby recycling nutrients and other essential materials 134. Autophagy is associated with both tumor suppression and progression depending on the tumor stage 135-140. The mTOR pathway has a decisive role in regulating autophagy, therefore targeting this pathway directly or indirectly through its upstream regulators helps in modulating the process 140. One of the main events used to confirm the occurrence of autophagy is the conjugation of microtubule-associated protein 1A/B light chain 3, LC3 I to LC3 II, which is incorporated into the complex degradation machinery 141. A study by Yue et al. shows a significant increase in protein levels of LC3 II accompanied by the inhibition of PI3K/AKT/mTOR pathway following the treatment of MG-63 osteosarcoma cells with DADS 142. Similarly, in myeloid leukemia cells, treatment with DADS decreases cell viability and increases apoptosis by inactivating the mTOR pathway 143. Moreover, the administration of NaHS effectively triggers protective autophagy in HCC cells by regulating the mTOR pathway 38. Collectively, these data identify the interaction of H_2_S donors with the mTOR signaling pathway as a crucial event in promoting autophagy and apoptosis thence preventing cancer progression.

#### H_2_S donor induces apoptosis

Apoptosis, a caspase-dependent programmed cell death, is amongst the highly investigated targets in cancer treatment. It helps to maintain normal cell metabolism by destroying specific cells that are not required without affecting their healthy neighbours 144. So far three major signaling pathways namely intrinsic, extrinsic 145, and perforin/granzyme B pathway 146 are known to induce apoptosis, all of which play an essential role in cancer modulation. Mitochondria, cell surface death receptors/adaptor proteins and the immune system are the main regulators for each pathway respectively. The activation of cysteine protease enzymes known as caspases is the common objective for all apoptotic pathways which result in the initiation and execution of proteolytic degradation by cleaving specific substrates such as poly adenosine diphosphate-ribose polymerase (PARP) and DNA fragmentation factor 147. Apoptotic pathways principally rely on pro- apoptotic caspase-2, -8, -9, and -10 for the initiation phase and caspase-3, -6, and-7 for the execution phase 148.

H_2_S donors regulate apoptosis in cancer cells by interacting with numerous apoptosis-inducing pathways 15, 149. The treatment with GYY4137 elevates the expressions of apoptotic markers caspase-9 and cleaved PARP in breast cancer MCF-7 cells, colorectal cancer Caco-2 cells 15, and ovarian cancer A2780 cells 150, with little or no effects in normal cell lines IMR90 cells 14 and Ea. hy926 150. STAT-3 pathway plays a crucial role in inducing apoptosis, and blocking this pathway is associated with improved apoptotic activities in different cancer types including lung cancer 149, pancreatic cancer 151, and HCC 152. Treatment with GYY4137 significantly increases apoptotic activities in HCC cells by preventing both interleukin-6 and JAK-2 induced phosphorylation of STAT-3 55.

Furthermore, treatment with NaHS upregulates the expressions of apoptosis-related genes caspase-3 and Bax and suppresses that of anti-apoptotic marker B cell lymphoma 2 (Bcl-2) by regulating p38 MAPK and p53 pathways 153. Likewise, treatment of human melanoma cells with ATB-346 promotes cell death by reducing the activities of cyclooxygenase-2 (COX-2) and inhibiting AKT and NF-кB signaling pathways 154. It has also been shown that the administration of ADT-OH can enhance anti-cancer activities in melanoma by reducing makorin ring finger protein 1 levels and preventing the degradation of IkBα, resulting in the accumulation of apoptotic adaptor protein Fas-associated protein with death domain and inhibition of NF-кB, respectively 155. In different cancer types, treatment with DATS has been shown to induce cell death by promoting mitochondria-mediated DNA damage 114 and by regulating AMPK 117, c-JUN N-terminal kinases (JNK) 119, PI3K/AKT 120, p38MAPK 156, and NF-кB 157 signaling pathways. Likewise, DADS treatment suppresses cancer progression by facilitating DNA damage 120 and inhibiting PI3K/AKT/mTOR 142, 143 and NF-кB pathways 32. In addition, compared to NaHS and GYY4137 treatments, breast cancer cells show extremely high apoptotic index when administered with HA-ADT (a conjugate formed with hyaluronic acid (HA) and ADT-OH) 12.

**Figure 3:**
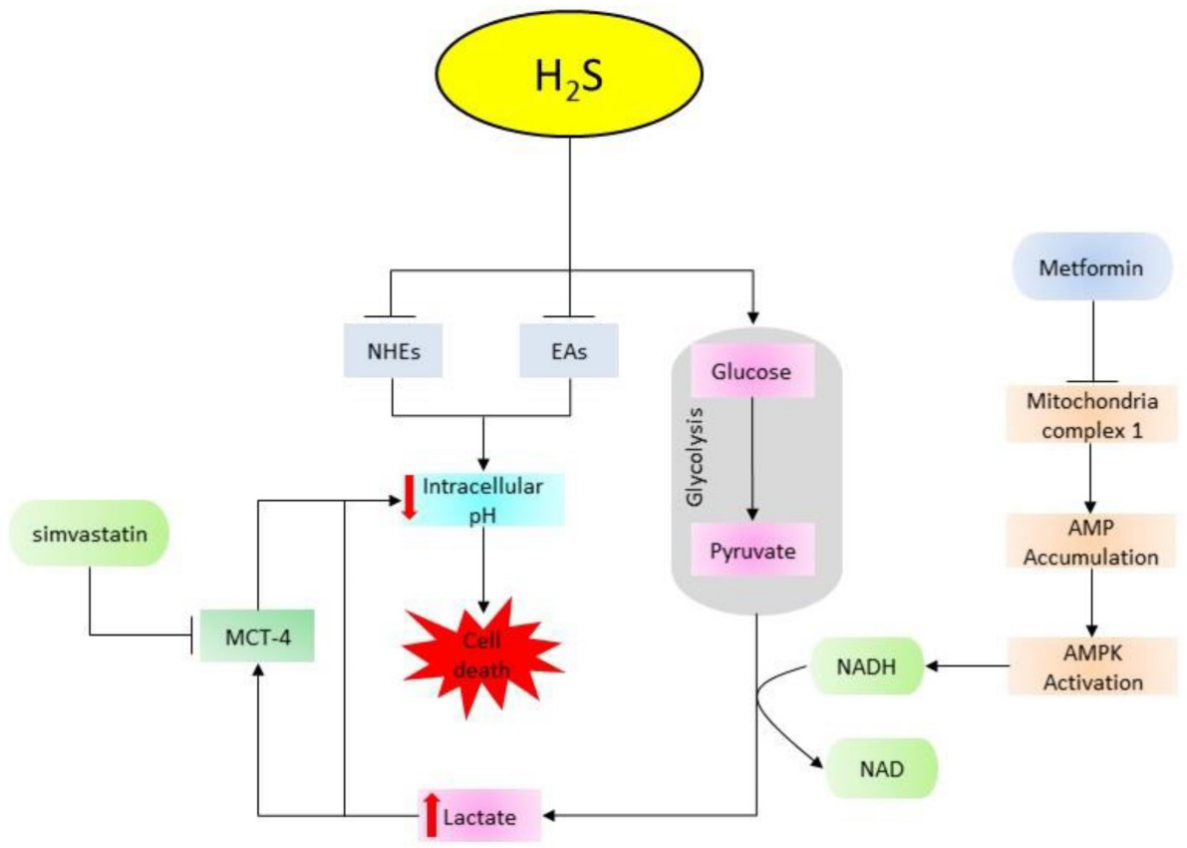
An illustration of the combination effect of H_2_S donors, metformin, and simvastatin in facilitating acidification-induced cell death. Metformin inhibits mitochondria complex 1, leading to the accumulation of AMP and the downstream activation of AMPK pathway. Next, AMPK increases NADPH which in turn facilitates the conversion of pyruvate into lactic acid thereby increasing intracellular acidity. H_2_S donors promote glycolysis and inhibit the activities of intracellular acidity regulators (NHEs and EAs). On the other hand, simvastatin prevents the exportation of lactate by inhibiting MCT-4. Together, all these processes cause cell death by decreasing intracellular pH. (AMPK: AMP-activated protein kinase; NADPH: nicotinamide adenine dinucleotide phosphate; NHE_S_: sodium/proton exchangers; AEs: anion exchangers; MCT-4: monocarboxylate transporter-4).

The extracellular acidic microenvironment is the key feature that differentiates cancer cells from non-cancer cells 158. The high rate of glycolysis in cancer cells results in the accumulation of lactic acid, leading to the reduction of intracellular pH (pHi) 159. Anion exchangers (AEs) and sodium/proton exchangers (NHEs) are key regulators of intracellular acidity recruited to adjust the decreasing pHi via active transportation of proton (H^+^) to the extracellular environment, making it more acidic compared to the intracellular environment 160, 161. A previous study indicates that low pHi enhances apoptosis through DNA fragmentation and activation of caspases 162. It has been revealed that treatment with GYY4137 increases intracellular acidity by promoting glycolysis and inhibiting the activities of NHEs and AEs 150, 163. However, compared to GYY4137, FW1010 (a structural analogue of GYY4137) promotes pHi-mediated cell death more efficiently 163. Moreover, synergizing GYY4137 with metformin (glycolysis-inducing type 2 diabetes mellitus drug) or simvastatin (a cholesterol/lipid-lowering drug targeting monocarboxylate transporter-4 (MCT-4)) aggravates the hyperacidity-induced cell death compared to treatment with individual drugs 164. This confirms that the interaction of donors with glycolysis in cancer suppression, and suggests a new direction of combination therapy comprising of H_2_S donors and glycolysis regulators (Figure 3). Overall, donor type and concentration play key roles in the regulation of apoptosis, however, slow-releasing donors and garlic-derived donors are more effective and efficient compared to the fast-releasing H_2_S donors. Regardless, their abilities to induce apoptosis by promoting intracellular acidification and activating both intrinsic and extrinsic apoptotic pathways suggest these donors can be used to target the majority of apoptotic pathways altered in cancer cells.

#### H_2_S donor enhances cancer drug sensitivity

Targeting cancer cells with more than one drug (combination therapy) that targets more than one pathway helps to reduce drug resistance and increase the efficiency of the treatment 165. The combination therapy can be chemotherapy and radiotherapy, chemotherapy and surgery, radiotherapy and surgery, or all three. In addition to their abilities to induce anti-cancer effects in drug resistant cancer cells such as cisplatin-resistant NSCLC A549/DPP cells 113, H_2_S donors improve the sensitivity and reduce resistance to anti-cancer drugs 118, 166. The downregulation of metallothionein 2A (MT2A), a stress protein is associated with poor prognosis in gastric cancer patients 167. It has been reported that DATS treatment can elevate both the mRNA and protein levels of MT2A, thereby improving the sensitivity of the anti-cancer drug, docetaxel (DOC), as well as the survival of gastric cancer patients 166. In human osteosarcoma cells, treatment with DATS reduces drug resistance by suppressing multidrug resistance protein 1 (P-gp1) 168. Furthermore, NaHS improves radiosensitivity in breast cancer by increasing tumor oxygen levels 23. NaHS also decreases hepatotoxicity induced by anti-cancer drug, methotrexate (MTX) 169. Collectively, cancer cells treated with donor-containing combination therapy demonstrate enhanced sensitivity and reduced drug resistance. Despite the need for further investigation, the data above indicate that H_2_S donors can regulate sensitivity and resistance-associated genes to enhance the anti-cancer activities of other drugs and further support their potential in cancer treatment.

#### H_2_S donor reduces tumor growth *in vivo*

*In vivo* xenograft tumor mice model is the most commonly used preclinical tool to determine the drug delivery system and response 170. Under the assessment, several parameters such as body weight, tumor mass, tumor volume, and protein expressions are analyzed and the results are compared with the control group. It has been shown that treatment of breast cancer tumor mice model with HA-ADT donor can effectively inhibit tumor growth and proliferation 12. Moreover, the treatment of leukemia xenograft tumor model with 100-300 mg/kg GYY4137 shows a significant reduction in tumor volume with no change in body weight or gross behaviour 14. GYY4137 donor (50 mg/kg/day) also reduces subcutaneous HepG2 tumor growth by regulating STAT-3 pathway 55. Besides, treatment with DADS/DATS inhibits *in vivo* tumor growth by reducing tumor size, weight, and regulating several factors involved in cancer progression 123, 130, 131. A study by Chandra-Kuntal, et al. also reports a significant reduction in tumor size and the downregulation of p-STAT-3 levels following the treatment of prostate cancer mice model with 2 mg DATS 171. In colon and gastric cancer mice model, DADS treatment promotes anti-cancer responses by reducing the level of vimentin and increasing that of E-cadherin 132, 133. Similarly, the treatment of HCC xenograft nude mice model with 0.8-1 mM NaHS can effectively inhibit tumor growth and progression 126. In summary, the evidence suggests that H_2_S donors have a dominant anti-cancer effect by reducing tumor volume, weight, growth, and regulating p-STAT-3 levels in a concentration-dependent manner.

### Cancer promoting activities

Despite the reported anti-tumor effect of H_2_S donors, on numerous occasions cancer promoting activities have been reported in different cancer cells when treated with H_2_S donors especially NaHS. Therefore, we further discuss the tumor promoting effects of these donors and the pathways involved.

#### H_2_S donor promotes cell cycle progression

The pro-carcinogenic effects of H_2_S are mainly induced by the facilitation of cell cycle progression and the activation of anti-apoptotic pathways. In oral squamous cell carcinoma, treatment with NaHS has been shown to facilitate cell cycle progression by downregulating the protein expressions of the DNA repairing RPA70 and tumor suppressor RB1, while upregulating those of the proliferating cell nuclear antigen (PCNA) and CDK4 172. In addition, the treatment decreases the expression of cell cycle-dependent kinase inhibitor 1 and activates the AKT/ERK 1/2 pathways 173. It has also been reported that treatment of multiple myeloma cells with NaHS reduces the proportion of cells in G0/G1 arrest and increases their proportion in S and G2/M phases 174. In summary, treatment with NaHS can stimulate cell cycle progression by amplifying the activation of AKT/ERK 1/2 signaling cascades.

#### H_2_S donor increases cell proliferation and viability

**Figure 4:**
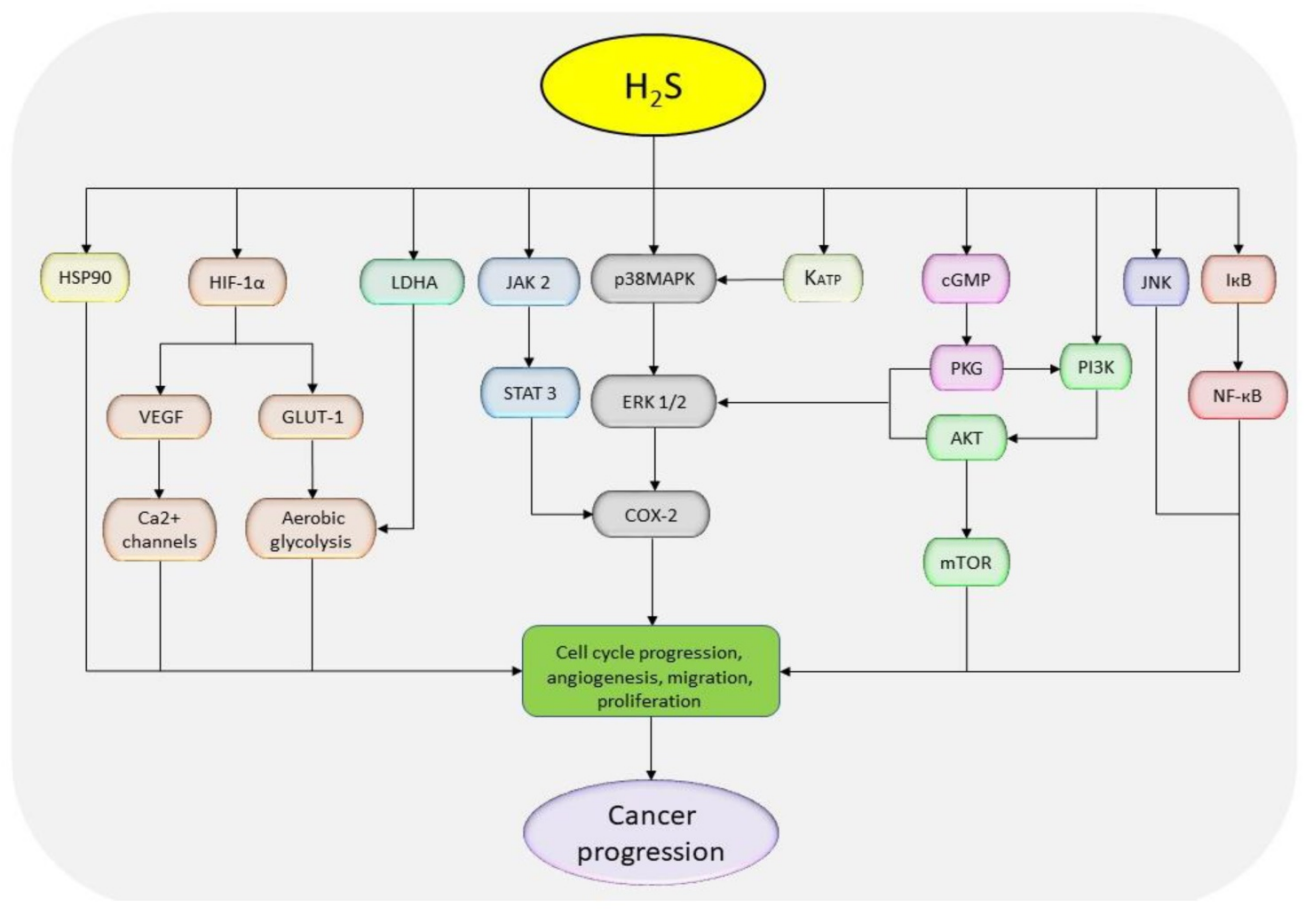
The diagrammatic presentation of cancer-promoting pathways regulated with NaHS treatment. H_2_S donors stimulate HSP90, HIF-1α, AKT, LDHA, JAK-2, MAPK, KATP, cGMP, and NF-қB, but inhibit JNK pathway to exhibit tumor promoting effects. (HSP90: heat shock protein 90; HIF-1α: hypoxia-inducible factor 1 alpha; AKT: protein kinase B; LDHA: lactate dehydrogenase A; JAK-2: Janus kinase 2; MAPK: mitogen-activated protein kinase; KATP: ATP-sensitive potassium channels; cGMP: cyclic guanosine monophosphate; NF-кB: nuclear factor-kappa B; JNK: c-Jun N-terminal kinase).

In addition to the promotion of cell cycle progression, studies also show that treatment with NaHS promotes cell proliferation and viability in esophageal carcinoma via the activation of heat shock protein 90 (HSP90) and JAK-2/STAT-3 pathways 18, 175. In HCC cells, NaHS treatment activates EGFR/ERK/MMP-2, PTEN/AKT, STAT-3/COX-2, and NF-кB and inhibits JNK signaling pathways (Figure 4) 126, 176, 177. Besides, the treatment of oral cancer cells (WSU-HN6, Tca83, and Cal27) with 1000 µM NaHS for 5 hours can enhance cell proliferation by activating COX-2/ERK/AKT pathways 178. However, conflicting results have been reported in C6 glioma cells where one study shows oncogenic activities induced via the activation of p38MAPK/ERK/COX-2 signaling cascades following 400-1600 µM NaHS treatment 179, while another study conducted on the same cell line (C6) using 250, 500, and 1000 µM NaHS shows anti-oncogenic effects accompanied by the activation of p38MAPK and p53 pathways 154. With the dose-dependent and fast-releasing nature of NaHS, the above confusion may be caused by the possible loss of H_2_S during the preparation of the samples and volatilization of the gas in culture plates, since the half-life of the compound is about 5 minutes of which over 80% of H_2_S is formed within the first 10 seconds after dissolving the crystals 180. Although, it's not clear how NaHS could result in stimulation of different MAPK isoforms, leading to the activation or deactivation of apoptotic pathway p53 181-183. In addition, it is not well understood as to why the effects of NaHS varies in different cancer types. Together, these data suggest that NaHS can impose pro-proliferating effects on cancer cells by promoting cell cycle progression and regulating the associated pathways.

#### H_2_S donor enhances cell migration and invasion

*In vitro* studies have reported that the incubation of endothelial cells with NaHS can significantly promote the migration and proliferation activities by stimulating HIF-α/VEGF, cGMP/protein kinase G as well as ATP-mediated potassium channels (K_ATP_)/p38 MAPK cascades 184-186. In breast tumor-derived endothelial cells, treatment with NaHS results in VEGF-mediated activation of Ca^2+^ channels and the promotion of cell migration 187. Besides, treatment of thyroid cancer cells with 25-50 µM NaHS effectively enhances migration and invasion by activating PI3K/AKT/mTOR and MAPK pathways 127. The H_2_S donor-mediated cell migration has also been confirmed by elevated levels of MMP-2 and -9 protein levels in bladder cancer and esophageal carcinoma following administration of NaHS 18, 175, 188. The cell migration-induced activation of AKT pathways have also been reported in multiple myeloma cells following the administration of NaHS 174. Meanwhile, in EC 109 and PLC/PRF/5 cells, the treatment signals were via HSP90 and NF-кB pathways, respectively 18, 176. These data suggest that treatment with H_2_S donors can intensify cancer metastasis through the regulation of PI3K, NF-кB, and MAPK pathways.

#### H_2_S donor suppresses apoptosis

The ability to suppress apoptotic activities is among the key features of cancer cells. Generally, compounds that increase cell apoptosis have great potential in therapeutic application. With respect to H_2_S donors, despite the observed potential, conflicting reports show a reduction in apoptosis following the treatment with NaHS. For instance, a previous study indicates that treatment with NaHS reduces the expression of apoptotic marker caspase-3 and Bax but increases that of Bcl-2 via the regulation of HSP90 18. Similarly, in multiple myeloma, NaHS treatment increases the expression of Bcl-2 and decreases that of caspase-3 via the stimulation of AKT signaling 174. In addition, it has been reported that NaHS treatment attenuates the expressions of caspase-9 and -12, and elevates those of VEGF and VEGFR in EC 109 cells 175. In brief, NaHS can interact with key cell regulators, resulting in enhanced pro-cancer effects.

#### H_2_S donor stimulates angiogenesis

The development and growth of new blood vessels from pre-existing ones is known as angiogenesis 189. It is a crucial process in tumor metastasis as it assures the supply of nutrients and other materials that are required for cell growth. In both normal and cancerous cells, angiogenesis is stimulated by VEGF proteins. A previous study suggests that H_2_S treatment can facilitate angiogenic responses such as microvessel sprouting of aortic ring via the regulation of H_2_S/NO signaling 186. It has also been reported that treatment with NaHS induces pro-angiogenic properties by overexpressing HIF-1α and downregulating micro RNA-640 via the vascular endothelial growth factor receptor 2-mTOR pathway 190. Similarly, a recent study shows that treatment of NSCLC with NaHS stimulates HIF-1α, resulting in VEGF activation and subsequently angiogenesis 191. Also, the treatment of EC 109 cells with 400 µm of NaHS promotes vascular formation by increasing the expression of VEGF and activating HSP 90 pathway 18. In brief, these data indicate that treatment with NaHS can promote cancer metastasis through the stimulation of angiogenesis which occurs via the stimulation of VEGF.

#### H_2_S donor modulates cellular bioenergetics

Cancer cells undergo several adjustments in energy supply mode so as to sustain the increased requirements. Cancer cells have been reported to undergo aerobic glycolysis in addition to the common mitochondria pathway in order to fulfill the high energy requirements 192. At lower H_2_S concentrations, most cells are reported to consume sulfide as a substrate in mitochondria sulfide-quinone reductase (SQR) of the mitochondria pathway, the event results in a reverse electron transfer in HT-29 colon cells 193. A recent study indicates that H_2_S signals by reprogramming energy metabolism which is in addition to the common pathway involving cysteine persulfidation 194. The study also demonstrates the dominant role of SQR in inhibiting the anti-proliferative properties of H_2_S at high levels. However, the involved mechanism is yet to be elucidated. Besides, it has been shown that the HIF-1α can positively mediate the functioning of glucose transporter-1 (GLUT-1), an important element in glucose transportation 195. On the downstream, the activation of GLUT-1 promotes the Warburg effect and reduces apoptosis 196. With respect to H_2_S, the activation of HIF-1α mediated by NaHS treatment has been reported in NSCLC 191. Therefore, it is possible that NaHS treatment could result in the elevation of cellular bioenergetics by enhancing HIF-1α and GLUT-1-mediated aerobic glycolysis. It has also been shown that the treatment of HCT116 colon cancer cells with low concentrations of GYY4137 increases mitochondrial function and glycolysis by persulfidating the Cys-163 of the lactate dehydrogenase A (LDHA) resulting in its stimulation 197. The activation of LDHA could result in increased glycolysis and Warburg effect 198. In summary, the above evidences indicate that H_2_S donors can induce pro-proliferative effects by modulating cellular bioenergetics via the activations of HIF-1α and LDHA.

## Conclusion

H_2_S donors have consistently been associated with the treatment of numerous cancer types and at different stages. These donors have shown great results in suppressing different cancer types by regulating the associated cellular activities and signaling pathways. However, the alarming inconsistency shown specifically in the case of NaHS (Table 1) needs to be further addressed. Currently, one of the explanations pinpointed is the short-time and high concentration-releasing nature of NaHS resulting in temporary elevation of cellular H_2_S levels followed by its subsequent decline which opposes the normal slow-releasing cellular mode and eliminate NaHS as a viable option in clinical settings. Furthermore, limited information on byproducts and clearance mechanism of these donors which raise many questions concerning their applicability. Therefore, it is important to determine the viable range of H_2_S donors' concentration and examine physiological factors such as toxicity and clearance mechanism. In addition, the effects imposed by the physical and chemical properties of these donors, their possible interactions need further investigation for better understanding of possible side effects. The combination of these donors with other cancer drugs such as docetaxel and cisplatin have presented improved drug sensitivity and reduced resistance 167, 168. Furthermore, the synergistic effects of H_2_S donors with metformin/simvastatin have been shown to promote anti-cancer activities by elevating intracellular acidity and impairing pH regulators. The combination of H_2_S donors with metformin/simvastatin or other cancer drugs might be useful in the treatment of advanced stages of cancer, and with the available data, it is worth conducting more animal studies and clinical trials to test the hypothesis. The effects of sulfuration-dependent post-translation modifications of proteins also need to be further investigated, since elevating the levels of H_2_S increases the chance of sulfuration which might affect the primary functions of numerous proteins. In conclusion, H_2_S donors have shown great potential in cancer treatment individually and in combination with other drugs, with more researches still ongoing, it is possible that these donors could be a great addition to cancer therapy, however more work is needed to be done.

## Figures and Tables

**Table 1:** The summary of the effects of NaHS in different cancer cell types.

Cancer types	Cell lines	NaHS doses	Effects on Cancer	References
Breast cancer	MCF-7	5-20 µM, 500 µM	Inhibition	14, 112
HCC	HepG2	5-20 µM, 1000 µM	Inhibition	14, 38
	HLE	1000 µM	Inhibition	38
	PLC/PRF/5	100-500 µM	Promotion	13
	SMMC-7721	10-100 µM	Promotion	126
	Huh-7	400-1000 µM	Inhibition	
Thyroid cancer	TPC-1 TT	10-50 µM	Promotion	127
	ARO	200 µM	Inhibition	
NSCLC	A549 A549/DDP	800 µM	Inhibition	113, 128
Esophageal carcinoma	EC 109	200-1000 µM	Promotion	18, 175
Glioma	C6	100-1600 µM	Promotion/Inhibition	153, 179
Gastric cancer	SGC 7901	200-800 µM	Inhibition	39
Oral SCC	GMN Cal-27 WSU-HN6	200-1000 µM	Promotion	172, 178
Multiple Myeloma	NCI-H929	250-1000 µM	Promotion	174
Bladder Cancer	EJ	400 µM	Promotion	188
Colon Cancer	HCT 116 SW480	50 - 200 µM	Promotion	173, 199
	SW480	1000 µM	Inhibition	

## Abbreviations

H_2_S: hydrogen sulfide
CBS: cystathionine beta-synthase
3-MPST: 3-mercaptopyruvate sulfurtransferase
CSE: cystathionine gamma-lyase
NaHS: sodium hydrosulfide
DAS: diallyl sulfide
DADS: diallyl disulfide
DATS: diallyl trisulfide
GYY4137: morpholin-4-ium-4-methoxyphenyl(morpholino) phosphinodithioate
ADT-OH: 5-(4-hydroxyphenyl)-3H-1,2-dithiole-3-thione
ATB-346: 2-(6-methoxynapthalen-2-yl)-propionic acid 4-thiocarbamoyl phenyl ester
ABCA1: ATP-binding cassette transporter A1
GLT-1: glutamine transporter-1
STAT-3: signal transducer and activator of transcription-3
NF-кB: nuclear factor-kappa B
Nrf-2: nuclear factor erythroid-2 related factor-2
HO-1: heme oxygenase-1
Tregs: regulatory T cells
TGF-ꞵ: transforming growth factor-beta
HCC: hepatocellular carcinoma
ROS: reactive oxygen species
PARP: poly adenosine diphosphate-ribose polymerase
eIF2α: eukaryotic translation initiation factor 2 subunit alpha
Keap 1: Kelch-like ECH associated protein 1
CDK: cyclin-dependent kinase
MMPs: matrix metalloproteinases
MT2A: metallothionein 2A
COX-2: cyclooxygenase-2
EGFR: epidermal growth factor receptor
ERK: extracellular signal-regulated kinase
Bcl-2: B cell lymphoma 2
Bax: Bcl-2-associated X protein
PI3K: phosphoinositide 3-kinase
Wnt: wingless integrated
ꞵ-catenin: beta catenin
AMPK: AMP-activated protein kinase
MAPK: mitogen activated protein kinase
cGMP: cyclic guanosine monophosphate
VEGF: vascular endothelial growth factor
SQR: sulfide-quinone reductase
LDHA: lactate dehydrogenase A
